# Future Liver Remnant (FLR) Increase in Patients with Colorectal Liver Metastases Is Highest the First Week After Portal Vein Occlusion: FLR Increase in Patients with CRLM Is Highest the First Week After PVO

**DOI:** 10.1007/s11605-018-4031-3

**Published:** 2018-11-21

**Authors:** Kristina Hasselgren, Per Sandström, Bård Ingvald Røsok, Ernesto Sparrelid, Gert Lindell, Peter Nørgaard Larsen, Anna Lindhoff Larsson, Nicolai A. Schultz, Bjorn Atle Björnbeth, Bengt Isaksson, Magnus Rizell, Bergthor Björnsson

**Affiliations:** 1 Departments of Surgery and Clinical and Experimental Medicine, Linköping University, Linköping, Sweden; 2 Department of Hepato-Pancreato-Biliary Surgery, Oslo University Hospital, Oslo, Norway; 3 Department of Clinical Science, Intervention and Technology, Division of Surgery, Karolinska Institutet, Karolinska University Hospital, Stockholm, Sweden; 4 Department of Surgery, Skane University Hospital, Lund, Sweden; 5 Department of Surgical Gastroenterology and Transplantation, Rigshospitalet, University of Copenhagen, Copenhagen, Denmark; 6 Department of Surgery, Akademiska University Hospital, Uppsala, Sweden; 7 Department of Transplantation and Liver Surgery, Sahlgrenska Academy, University of Gothenburg, Gothenburg, Sweden

**Keywords:** Colorectal liver metastases, Liver surgery, Future liver remnant, Portal vein embolization, Portal vein ligation

## Abstract

**Background:**

Portal vein occlusion (PVO) is an established method to increase the volume of the future liver remnant (FLR). The main reasons for not proceeding to radical hepatectomy are lack of volume increase and tumor progression due to a wait-time interval of up to 8 weeks. The hypothesis was that the increase in FLR volume is not linear and is largest during the first weeks.

**Methods:**

Patients with colorectal liver metastases (CRLM) and standardized future liver remnant (sFLR) < 30% treated with PVO were prospectively included. All patients had at least one CT evaluation before radical hepatectomy.

**Results:**

Forty-eight patients were included. During the first week after PVO, the kinetic growth rate (KGR) was 5.4 (± 4), compared to 1.5 (± 2) between the first and second CT (*p* < 0.05). For patients reaching adequate FLR and therefore treated with radical hepatectomy, the KGR was 7 (± 4) the first week, compared to 4.3 (± 2) for patients who failed to reach a sufficient volume (*p* = 0.4). During the interval between the first and second CT, the KGR was 2.2 (± 2), respectively (± 0.1) (*p* = 0.017).

**Discussion:**

The increase in liver volume after PVO is largest during the first week. As KGR decreases over time, it is important to shorten the interval between PVO and the first volume evaluation; this may aid in decision-making and reduce unnecessary waiting time.

## Introduction

Portal vein occlusion (PVO) (portal vein embolization (PVE) and portal vein ligation (PVL)) is an established method for patients with inadequate volume of the future liver remnant (FLR) who require major liver resection. When indicated, PVE is technically feasible in up to 98% of the patients and with a median increase of the FLR of up to 50%.^1^–^3^

The exact mechanisms behind the increase of the FLR after PVE and PVL are not known. Some of the factors associated with reduced increase are liver cirrhosis, procedure-related complications, elevated bilirubin before PVO, obesity, male gender, and diabetes.^3^,^4^ Data regarding the impact of preoperative chemotherapy on the growth of the FLR after PVE are conflicting, although most studies report any impairment of the FLR increase in patients with pre-procedural chemotherapy. Others report impaired hypertrophy, especially after more than six cycles of chemotherapy or after administration of bevacizumab.^5^–^8^

The increase of the FLR after PVL is similar to PVE, although data exist that indicate volume expansion may be smaller after PVL.^9^,^10^

PVO can be performed either in conjunction with two-stage hepatectomy (TSH), where the FLR is cleared of tumor before induction of growth, or prior to one-stage hepatectomy. The increase of the FLR volume is similar with these two approaches. The majority of patients who do not proceed to hepatectomy are failing due to tumor progression.^11^

The prognosis and long-term survival after two-stage hepatectomy (TSH), including PVO in patients with colorectal liver metastasis (CRLM), is comparable to patients treated with one-stage hepatectomy without prior PVO, with a 5-year overall survival above 40%.^12^–^14^

The recommended interval between PVO and assessment of the FLR is commonly between 4 and 6 weeks, causing the interval between PVO and hepatectomy to be a median of 50 days, although the interval was reported to be just over 2 weeks in early reports of patients treated with hepatectomy after PVE.^13^–^16^

However, 25 to more than 40% of patients treated with PVO fail to qualify for radical hepatectomy, either due to insufficient growth of the FLR or to tumor progression during the long waiting period.^11^,^17^

Experimental and clinical studies indicate that hypertrophy may be more pronounced in the early period after PVO. The data is sparse, especially for the first and second week after PVO.^18^,^19^ The introduction of associating liver partition and portal vein for stage hepatectomy (ALPPS) has clearly shown that the human liver has the capacity of rapid hypertrophy with growth rate that exceeds observed growth rate after PVO. In the settings of ALPPS, previous studies indicate that the increase in sFLR volume the first week after stage 1 is higher than 50%.^20^,^21^

If the FLR growth rate is indeed fastest in the early period after PVO, CT evaluations made within 2 weeks following PVO could potentially identify patients reaching adequate FLR volume earlier, thereby lowering the risk of tumor progression in the waiting period. Furthermore, patients with very slow initial FLR growth rate could be identified, allowing consideration of an early strategy change.

The hypothesis for this study was that liver hypertrophy after PVO is more pronounced during the first weeks after the intervention compared to later in the course before radical hepatectomy. Early evaluation may identify patients showing inadequate FLR increase and thus allow the planning supplementary techniques for volume expansion of the FLR. In addition, patients reaching adequate FLR volume may be offered radical hepatectomy earlier than previously thought.

The aim of this study was to study the kinetics of growth of the FLR until the final decision was made for liver resection or failure to resect, with a special focus on the growth rate during the first week after PVO.

## Material and Methods

This study analyses patients treated with PVO within a Scandinavian multicenter randomized trial (ClinicalTrials.gov, NCT02215577).^22^

Inclusion criteria were potentially resectable colorectal liver metastases with an insufficient sFLR (less than 30%), thus requiring preoperative volume expansion. Exclusion criteria were liver cirrhosis, significant comorbidity, and/or age less than 18 years.

All participating centers were specialized HPB centers with a long experience of portal vein occlusion. The method used at each center was consistent during the study period.

All patients with metastases in the FLR were treated with either local resection and/or ablation of the metastases in the FLR. Patients treated with PVE had the ablation or local resection in conjunction with the PVE or the radical hepatectomy, and patients treated with PVL had the ablation or local resection in conjunction with the PVL.

Volumetric evaluation was performed within the first week after PVO and for those with inadequate increase of the FLR within an additional 2 or 4 weeks, depending on the volume of the FLR at first evaluation and KGR during the preceding period. sFLR was calculated from a previously described formula.^23^ Degree of hypertrophy (DH) was calculated as the difference in sFLR between two time points: DH = post-PVO sFLR – pre-PVO sFLR. KGR was calculated by dividing DH with the time between the measurement points, expressed in weeks: KGR = DH ∕ number of weeks.^19^ KGR was calculated for the intervals between PVO and the first computed tomography (CT) evaluation, for the intervals between eventual complementary evaluations separately and between the PVO and last evaluation before radical hepatectomy or exclusion.

Patients treated with PVE and PVL were analyzed together.

Further details about the material and methods of the study have been published earlier.^22^

Ethical approval was obtained in all participating countries. The ethical review boards from each participating country (Sweden, Denmark, and Norway) approved the trial. Written informed consent was obtained from each patient enrolled.

## Statistical Analyses

The results are expressed as the mean ± standard deviation or median (range), as appropriate. KGR is expressed as %/week. Continuous data were compared with *t* test or ANOVA. Post hoc analyses were performed using the Bonferroni correction. Categorical data was compared with a chi-squared test.

Analyses were performed using IBM SPSS (version 23; IBM Corp, Armonk, NY, USA). A *p* value less than 0.05 was considered significant.

## Results

Fifty patients included in the LIGRO trial were randomized to PVO. One patient was excluded due to advanced disease at the time of inclusion, and one patient was excluded due to missing volumetric data. Forty-eight patients thus were analyzed. Thirty-four patients (71%) were treated with PVE and 14 patients (29%) received PVL. Thirty patients (63%) had metastases in the FLR, of which 14 were treated with PVL and 16 (46%) were treated with PVE. Additional clinical variables are found in Table 1.

**Table 1:** Clinical variables and preoperative data

Number of patients	48
Male/female	36/12
Age (mean ± 1 SD)	65 ± 12
BMI (mean ± 1 SD)	26 ± 4
ASA (median, range)	2 (1–3)
ECOG (median, range)	0 (0–1)
Cortisone treatment	1 (2%)
Diabetes mellitus	6 (13%)
Primary tumor resected/not resected	30/18
Chemotherapy cycles (mean ± 1 SD)	7 ± 4
Response to chemotherapy/stable disease	37/9*
Time (days) between last dose of chemotherapy and first intervention (median, range)	35 (7–412)
Liver metastases at time of surgery (median, range)	8 (1–23)
Size (mm) of the largest liver metastasis (mean ± 1 SD)	48 ± 38
Metastases in the FLR	30 (63%)
Extra hepatic disease	7 (15%)
Local lymph node enlargement	2 (4%)
Complications after PVO	8 (17%)**

*One patient did not receive chemotherapy and for one patient, the response to given chemotherapy was difficult to evaluate. ** Data are lacking for one patient. Complications were grade 1 or 2 according to the Clavien-Dindo classification

Five patients had only one CT evaluation before radical hepatectomy, and forty-three patients had additional radiological evaluation before radical hepatectomy or exclusion due to insufficient increase of the volume of the FLR or due to tumor progression.

Twenty-seven patients (56%) had sufficient FLR increase, resulting in a resection rate of 56% after PVO. Twenty-one patients failed to qualify for radical hepatectomy; 14 patients had insufficient FLR increases, and seven patients had tumor progression.

For the entire cohort, the pre-PVO sFLR was 21% (± 5). The KGR was 2.8 (± 2) between PVO and the last radiological evaluation before determining the final outcome, which was either radical hepatectomy or exclusion due to failure to increase the volume of the FLR or tumor progression. For the group who proceeded to radical hepatectomy, the KGR was 3.4 (± 2) between PVO and radical hepatectomy. For patients who failed to reach the sufficient volume of the FLR, the KGR was 1.5 (± 1) from PVO until the last CT evaluation. The difference was statistically significant (*p* = 0.015). The median time from PVO until the final CT before radical hepatectomy/exclusion was 4 weeks (1–11). Radical hepatectomy was performed 6 weeks (4–14) after PVO.

**Fig. 1:**
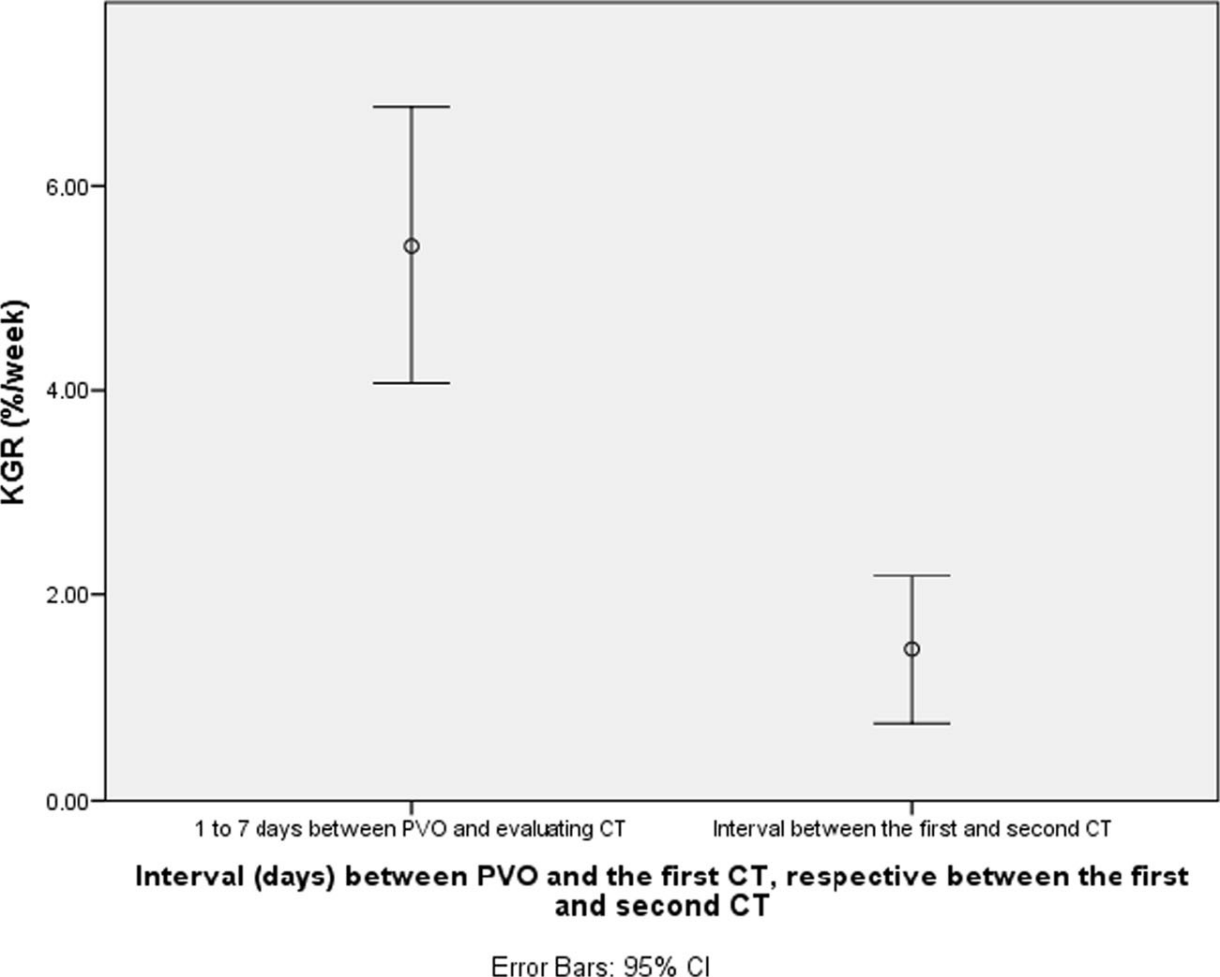
Temporal increase of the FLR, expressed as KGR, from PVO to the first evaluation and between the first and second CT. The KGR was 5.4 (± 4), compared to 1.5 (± 2) between the first and second CT. The difference was statistically significant (*p* < 0.05). The interval between the first and second CT was between 7 to 35 days

During the first 7 days after PVO, the KGR was 5.4 (± 4), compared to 1.5 (± 2) between the first and second CT (*p* < 0.05; see Fig. 1). Thirty patients did the first CT within 7 days from PVO, and 13 were within 8 to 15 days. Twenty-three patients had the second CT within 3 weeks, and 11 patients received CT after more than 3 weeks.

When the interval between PVO and first evaluation CT was stratified to periods of 1 to 7 and 8 to 15 days, KGR in the group evaluated within 7 days was found to be 5.4 (± 4) while for the patients evaluated between 8 and 15 days, the KGR was 3.8 (± 2) (*p* = 0.54).

**Fig. 2:**
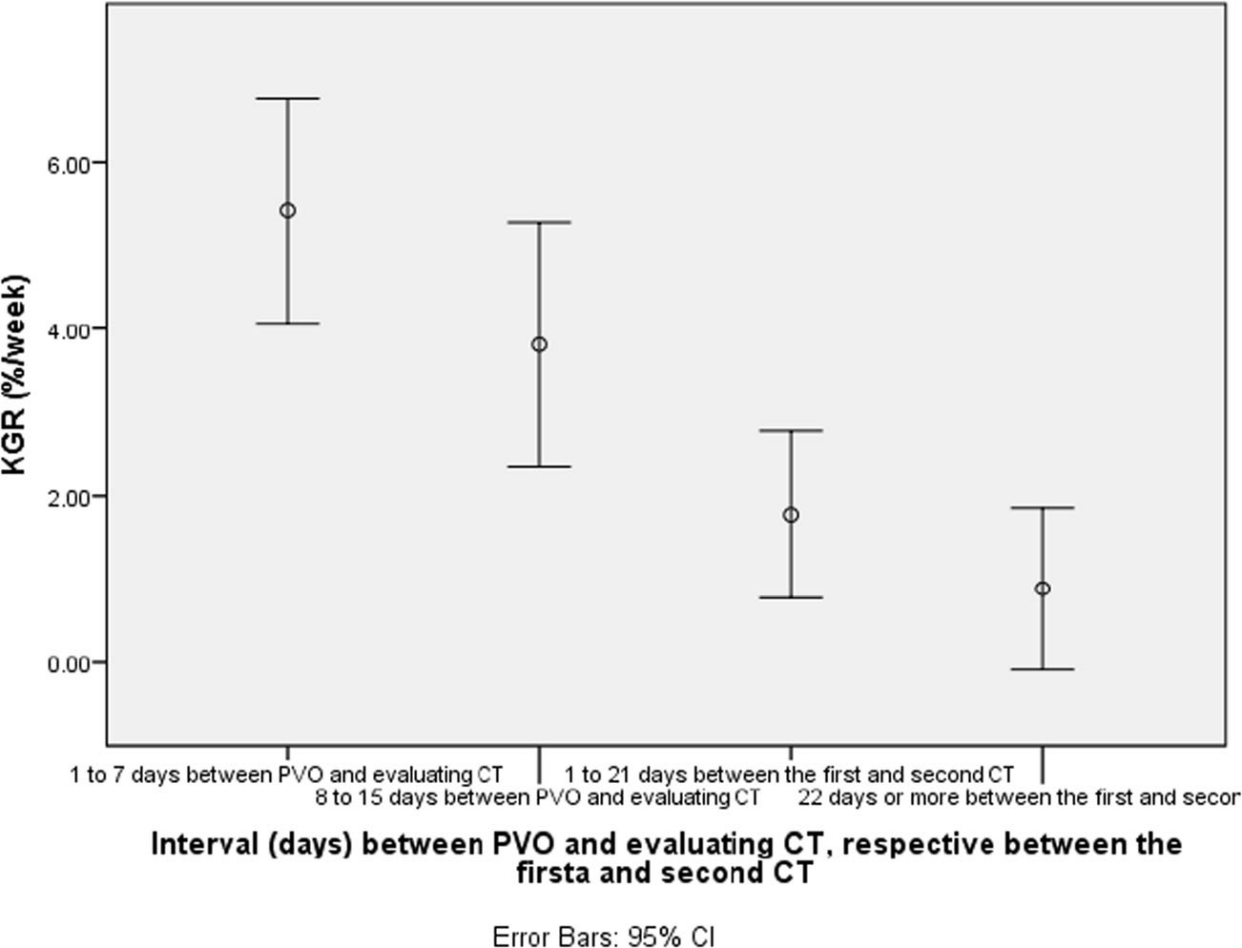
Temporal course of the increase of FLR, with the intervals 1 to 7 days between stage1/PVE and the first CT, and 1 to 21 and 22 days or more between the first and second CT. The KGR was 5.4 (± 4), 3.8 (± 2), 1.8 (± 2), and 0.9 (± 1). The difference between KGR values for the interval of 1 to 7 days compared to the intervals of 1 to 21 days and 22 days or more between the first and second CT evaluation was statistically significant at *p* < 0.005

Further analyses of the interval between the first and second CT, divided in 1 to 21 days and 22 days or more, revealed that the KGR was 1.8 (± 2) and 0.9 (± 1), respectively (*p* = 0.165; see Fig. 2).

A comparison between the groups that proceeded to radical hepatectomy, those who failed to achieve sufficient volume and those with tumor progression is shown in Table 2. Comparing the patients who proceeded to the radical hepatectomy with those who failed to gain sufficient volume after PVO revealed that during the first 7 days, the KGR was 7 (± 4) and 4.3 (± 2), respectively. Although the latter group had a lower rate of hypertrophy during this interval, the difference did not reach statistical significance (*p* = 0.4). However, during the interval between the first and second CT evaluation, KGR was 2.2 (± 2) and 0.1 (± 0.9), respectively (*p* = 0.017).

**Table 2:** Volumetry for the entire cohort and analysis of the subgroups success, failure, and tumor progression

Volumetry	Entire PVO cohort (*n* = 48)	Treated with radical hepatectomy (*n* = 27)	Failure to achieve sufficient volume (*n* = 14)	Tumor progression (*n* = 7)	*p* value
sFLR pre-PVO (percent of standardized FLR)	21 ± 5	23 ± 4	17 ± 5	22 ± 5	< 0.05*
FLR pre-PVO (ml)	365 ± 105	415 ± 95	281 ± 77	331 ± 52	< 0.05*
KGR day 1–7 after PVO (%/week)	5.4 ± 4	7 ± 4	4.3 ± 2	2.6 ± 0.8	Ns
KGR between the first and second CT (%/week)	1.5 ± 2	2.2 ± 2	0.1 ± 0.8	2.4 ± 1	0.017*
Increase in ml from PVO to the last evaluation (ml)	156 ± 118	217 ± 123	79 ± 41	77 ± 39	< 0.05* 0.004**
Increase in percent from PVO to the last evaluation (%)	43 ± 30	55 ± 33	31 ± 21	24 ± 13	0.044* 0.043**
sFLR at last volume assessment (percent of standardized FLR)	30 ± 10	35 ± 9	21 ± 5	27 ± 6	< 0.05* 0.043**
Days between PVO and the first CT evaluation	10 ± 8	11 ± 9	9 ± 8	8 ± 3	Ns
Days between the first and second CT evaluation	22 ± 9	23 ± 12	23 ± 6	17 ± 5	Ns

*Comparison of success and failure. **Comparison between success and progress. Data are presented as the mean ± 1 SD

Of the patients who proceeded to radical hepatectomy, eight patients (29%) reached sufficient volume of the FLR within 7 days of PVO, five patients (18%) reached sufficient FLR volume within 15 days, and two patients (7%) reached sufficient FLR volume within a month of PVO.

The increase in FLR, expressed as either increase in milliliters or percent, did not differ significantly between patients treated with PVE or PVL.The KGR during the first week was higher for patients treated with PVL compared to PVE, at 7.0 (± 5) and 4.3 (± 2), respectively (*p* = 0.043). The difference in KGR between the first and second CT evaluation did not differ significantly, being 1.9 and 1.3. There was no significant difference in the number of patients treated with radical hepatectomy and failure to increase the FLR or tumor progression between PVE- and PVL-treated patients.

The pre-procedural sFLR for those who proceeded to radical hepatectomy was 23% (± 4), compared to 17% (± 5) in the group who failed to reach sufficient FLR volume, but the difference did not reach statistical significance. For the subgroup of patients who reached sufficient volume of the FLR within 15 days of PVO, the pre-procedural sFLR was 22% (± 5), compared to 24% (± 4) for those who reached sufficient volume after more than 15 days after PVO. The difference was not statistically significant.

Patient-related factors, such as pre-PVO bilirubin and BMI, did not significantly correlate to KGR. No significant difference was found between male and female patients. There was no difference in the number of cycles of chemotherapy between the group who reached sufficient volume and to the group who did not. There was no correlation between the number of cycles of chemotherapy and KGR.

## Discussion

This is the first study to show that the largest hypertrophy after PVO occurs during the first week and, furthermore, that the kinetic growth rate of the liver is higher when evaluated after only 1 week than it is when the evaluation is postponed beyond 7 days. Accordingly, the increase of the FLR volume is not linear over time after PVO but instead is largest during the first week.

There are few previous studies in which the growth kinetics after PVO has been analyzed, typically within the first few weeks post-intervention. In one study, patients who failed to proceed to surgery due to tumor progression were followed with sequential CT scans for up to nearly a year, demonstrating continued increase in FLR volume during the entire follow-up period. However, the increase was largest during the first 3 months after PVE. The results are not entirely comparable with the results of the present study because the intervals are different, and the increase is measured as a percent (absolute increase) and not KGR (increase over time).^24^ These results also differ somewhat from another previous study, in which the FLR increased more during a follow-up period of 6 months than during the first 23 days after PVE. It is nevertheless likely that KGR would be larger in the first few weeks if it had been calculated in that study.^25^

The results of this study are coherent with the findings from MD Anderson Cancer Center, where KGR (when measured during the entire hypertrophy period) was shown to be lower for patients who needed prolonged time before hepatectomy compared to those who were able to proceed to early surgery.^19^

The number of patients in this study is limited, and not all patients were examined with repeated CT between PVO and radical hepatectomy, which reduces the statistical power of the study. Another limiting factor that must be considered is that the method for PVE and volume measurement differed between the participating centers. However, methods were consistent at each center. However, none of the different materials used as embolization agents, such as gelatin sponge, alcohol, polyvinyl alcohol (PVA) particles, and *N*-butyl cyanoacrylate (NBCA) glue, have been clearly demonstrated as being superior to another terms of efficacy to increase the FLR volume.^26^,^27^

There are several different methods to study liver function, but none of those has been found to be more accurate than the other. Controversies exist regarding whether the increase of volume corresponds to an equal increase in liver function or not. Some data based on hepatobiliary scintigraphy indicate that the volume of the FLR after PVE does not correlate with function,^28^,^29^ while this has not been studied in the immediate period after PVO, leaving an important question unanswered about the quality of early hypertrophy. The function of FLR was not evaluated in this study. Furthermore, it is important to recognize that in this study, the definition of failed hypertrophy was based on the final assessment volume of the FLR, while KGR previously has been shown to have prognostic value for post-hepatectomy liver failure.^19^

Despite these limitations, this study clearly demonstrates a large and significant difference in hypertrophy rate that decreases over time after PVO. This new information, together with the knowledge that failure to complete hepatectomy may be due to inadequate hypertrophy or disease progression, makes it important to identify variables that may indicate early in the process if hypertrophy will be sufficient. Comparing early KGR directly between those with and without adequate hypertrophy did not significantly discriminate between the groups. As reported earlier, patients who failed to reach a 30% sFLR had lower pre-procedural sFLR, and none of the patients with an initial sFLR below 17% completed radical hepatectomy.^22^ These data indicate that patients with very low initial sFLR are at risk of not achieving sufficient FLR volume.

The main reason reported for patients not proceeding to radical hepatectomy after PVO is tumor progression following the initial intervention. However, in this study, we found a higher proportion of patients with insufficient growth. A probable explanation for this, is that the study protocol demanded sFLR > 30% for proceeding to radical hepatectomy and had a lower limit of volume increase (KGR > 2%/week) per week. For patients who require a longer period of time between the first and second stage to gain sufficient FLR volume, administration of chemotherapy may reduce the risk of tumor progression. The risk also may be reduced with a shorter interval for the patients that reach a sufficient volume.^30^,^31^ The results from this study show that half of the successfully treated patients reached sFLR greater than 30% at the first radiological evaluation, indicating that radical hepatectomy may, at least for some patients, be performed earlier than after the traditional 4- to 6-week waiting period.^22^

It is reasonable to evaluate the increase of the FLR with shorter intervals immediately after PVO, compared to a standard CT evaluation 1 month after the intervention.^19^ A relatively large proportion of the patients reached sufficient volume of the FLR after the first week, allowing early radical hepatectomy.^22^ Furthermore, the findings in this study indicate that patients with low initial KGR, and especially with very low initial FLR, may be at risk for failure of hypertrophy and should therefore be reassessed early in order to allow for other methods to increase the FLR volume when needed.

## Conclusion

Volume expansion of the FLR in patients with resectable colorectal liver metastases is largest during the first week following PVO. It is important to perform the first evaluation after PVO shortly after the first week in order to individualize the treatment and avoid unnecessary delay of treatment.
